# Gold-Nanoparticle-Enhanced Radio-Fluorogenic Hydrogel Sensor for Low Radiation Doses in Clinical Radiotherapy

**DOI:** 10.3390/polym14224841

**Published:** 2022-11-10

**Authors:** Xingyu Dong, Yuan Tian, Fengqing Wang, Chong Chen, Yunlong Wang, Jun Ma

**Affiliations:** College of Materials Science and Technology, Nanjing University of Aeronautics and Astronautics, Nanjing 211106, China

**Keywords:** radio-fluorogenic hydrogel, gold nanoparticles, radiation dosimetry, radiotherapy

## Abstract

Radio-fluorogenic hydrogel dosimeters are urgently needed in radiotherapy for 3D dose verification. However, few hydrogel sensors have been reported at low absorbed doses under 2 Gy which meets the requirements of clinical practice. Here, we report a new type of gold-nanoparticle-enhanced radio-fluorogenic agarose hydrogel with coumarin as the dose-responsive material. An optimal composition of 3 wt% of agarose, 0.1 mM of gold nanoparticles, and 0.5 mM coumarin was selected. The addition of gold nanoparticles enhanced the hydroxyl radicals generated from the radiolysis of water, which can react with coumarin and generate fluorescent 7-hydroxy-coumarin and, eventually, achieve low-dose verification of 0–2.4 Gy with a high linear correlation coefficient. These findings provide an effective method for 3D dose verification, and will inspire the development of other radio-fluorogenic sensing hydrogels as well.

## 1. Introduction

Cancer mortality is showing an upward curve due to low prognosis and rapid metastasis. Current clinical treatments for cancer include chemotherapy, immunotherapy, surgery, and radiotherapy [1]. Radiation therapy is one of the most commonly used cancer treatment methods. It uses high-energy ionizing radiation (X-ray or e-beam) to pinpoint and kill cancer cells, and eliminate the tumor, by introducing DNA damage in tumor tissue [2]. In radiotherapy, dosage verification can effectively reduce the number of errors caused by the planning software [3], hence decreasing the occurrence of radiation sickness. Dose verification in clinical radiotherapy usually includes point dose verification [4,5,6], surface dose verification [7,8], and three-dimensional dose verification [9]. In these methods, only three-dimensional dose verification can accurately reflect the stereoscopic distribution of absorbed doses in the target tissue during radiotherapy. Therefore, it has attracted increasing focus over the last two decades, and is believed to be the future of dose verification. Hydrogels possess tissue-equivalence and the potential to be changed into pre-specified 3D shapes to meet the anatomical characteristics of the human body [10,11,12], thus making it the best candidate for three-dimensional dosimetry dosimeters in clinical dosimetry verification. In recent years, many works have been reported to prepare hydrogel dosimeters with high sensitivity, accuracy, and spatial resolution, as well as independence of beam type, energy, and dose rate [13]. However, challenges still exist in the application of hydrogel dosimeters for low-dose range verifications.

Existing research has mainly concentrated on dosimeters based on radiation oxidation/reduction of metal ions, such as Fricke, or ferrous–benzoic–xylenol (FBX) hydrogel dosimeters [14,15,16,17,18,19,20,21,22]. At present, Fricke gel dosimeters are greatly hampered by their diffusivity and lack of detection capability for low doses in clinical applications [16,23]. A gel dosimeter using a reduction of HAuCl_4_ has been reported by Inamdar et al., and enables dose measurement in the range of 0–2 Gy as well as dose visualization [18,21]. However, these hydrogel dosimeters cannot be preserved for an extended period of time. Another popular product is the radiochromic hydrogel dosimeter. These dosimeters have the advantages of simple operation, no need for developing, as well as direct dose visualization; however, these sensing gels have poor tissue-equivalence [24,25]. Radiation polymerization hydrogel sensors need to be scanned by CT to visualize the absorbed doses, which is a complicated and expensive procedure [26,27]. In recent years, fluorescent hydrogel sensing materials have been proposed [28] which have the benefits of long-term storage stability, good tissue equivalency, and simplified, low-cost visualization in comparison to previously mentioned sensing hydrogels. Coumarin (COU) has an ability to respond to radiation exposure, as it can be converted into fluorescent 7-hydroxy-coumarin (7-HCOU) by the hydroxyl radical (·OH) caused by water radiolysis [29,30,31]. Due to its high solubility in aqueous solution, simple chemical composition, and suitable excitation/emission spectra, it has been seen as one of the potential sensing reagents for radiation dose verification. For instance, coumarin 3-carboxylic acid solutions have been employed as liquid radiation sensors [32,33], demonstrating good dose-response and long-term stability. Peter A. Sandwell et al. developed a hydrogel sensor with gelatin as the matrix and coumarin 3-carboxylic acid as the fluorescent response material, and investigated its radiation dosimetric characteristics [34]. However, with a responsive range from 20–50 Gy, the sensitivity of these hydrogels was not sufficient for the measurement of low dosages (0–2 Gy) which is the range required by clinical fractionated radiotherapy.

Gold nanoparticles (GNPs) have been used to enhance radiation damage to cancer cells [35,36,37]; this enhancement is considered to be caused by the increased yield of ·OH in the radiolysis of water [38,39,40]. Thus, the addition of GNPs can increase the yield of 7-HCOU and increase fluoresce intensity after exposure to radiation. As previously reported in the literature [17], agarose is a functional polysaccharide, and the incorporation of GNPs in agarose gels facilitated the templating of GNPs. As a common biomedical gel material, agarose has the benefits of biocompatibility and ease of preparation, meeting the clinical requirements of non-toxicity and convenience. At the same time, agarose, as a single hydrogel, does not react with COU, which is the dose-responsive material in our hydrogel sensor. Meanwhile, the mechanical properties of this hydrogel are easily adjustable. Therefore, we chose agarose hydrogel as the substrate material for this hydrogel sensor.

Here we report for the first time a highly sensitive GNP fluorescent hydrogel sensor for low-radiation-dose measurements, which was created by combining GNPs with coumarin as a responsive material and agarose as a substrate. This high-sensitivity hydrogel sensor is proven to emit enhanced fluorescence after being irradiated, and can work as a more-sensitive radiation dosimeter that can satisfy the measurement of dose-response up to 2 Gy. With the advantages of high sensitivity, accurate measurement, and biocompatibility, this radiation-sensing hydrogel can greatly benefit three-dimensional dose verification in clinical fractionated radiotherapy.

## 2. Materials and Methods

### 2.1. Materials

Coumarin (98%, AR) was purchased from Macklin Technology Co., Ltd., Shanghai, China, agarose (gel strength 1%) was purchased from Labgic Technology Co., Ltd., Hefei, China, HAuCl_4_·4H_2_O (AR) was purchased from Sinopharm Chemical Reagent Co., Ltd., Shanghai, China, Sodium citrate (99%, AR) was purchased from Sigma-Aldrich Co., Ltd., Japan, Commercial GNPs (0.1 mg/mL) in sizes of 30 nm, 50 nm, and 70 nm were purchased from Beijing Zhongkeleiming Daojin Technology Co., Ltd., Beijing, China, and 96-well solid white polystyrene assay plates (Costar 3915) were purchased from Corning Inc. Shanghai, China, MilliQ water was used as solvent for all experiments conducted.

### 2.2. Methods

#### 2.2.1. Preparation of GNP Solution

A 199 mL volume of tetrachloroauric acid (0.25 mM in MilliQ water) was added into a round-bottom flask and stirred at 100 °C for 15 min. After that, 1 mL of sodium citrate solution (500 mM in MilliQ water) was quickly added to the round-bottom flask and continued to react at 100 °C for 30 min, and eventually, a wine-red GNP solution was obtained [41]. The size of the GNPs in the synthesized GNP solution was determined to be about 20 nm by DLS (dynamic light scattering), TEM (transmission electron microscopy), and UV absorption peak positions of GNPs (Figure S1, Supporting Information).

#### 2.2.2. Preparation of Hydrogel Sensor

First, COU was dissolved in MilliQ water to obtain the final concentration of 25 mM and heated until a clear transparent solution was obtained at 95–100 °C. Agarose, COU solution (25 mM), and GNP solution (0.25 mM) were dissolved in 10 mL of water and filled in a 25 mL volumetric flask; the final content of agarose, COU, and GNP in the solution were 3 wt%, 0.5 mM, and 0.1 mM, respectively. Then, the mixed solution was heated to 95 °C and stirred in a water bath for 15 min to obtain a transparent ruby color. Finally, the hot mixed liquid was added to the 96-well black cell plate to form the sensing hydrogel.

#### 2.2.3. Dose-Response of Hydrogel Sensor

**Figure 1:**
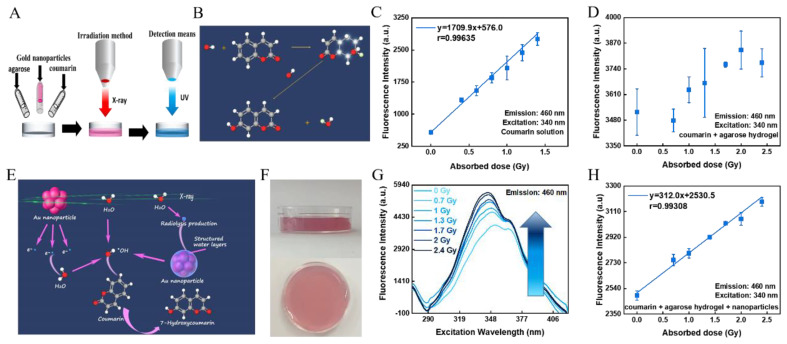
Dose-response and principles of hydrogel sensors. (**A**) Flow diagram of the preparation and irradiation process of a hydrogel sensor. (**B**) Schematic representation of the reaction of ·OH with COU to form 7-HCOU. (**C**) Fluorescence intensity of COU solution (0.5 mM) being irradiated for different doses. (**D**) Fluorescence intensity of mixed gel of COU (0.5 mM) and agarose (3 wt%) being irradiated for different doses. (**E**) Schematic diagram of the pathway of ·OH production and 7-HCOU in a hydrogel sensor containing GNPs. (**F**) Top and front view of the hydrogel sensor. (**G**) Fluorescence intensity with different excitation wavelengths of the hydrogel sensor containing GNPs. (**H**) Fluorescence intensity of agarose (3 wt%) hydrogel sensor containing GNPs (0.1 mM) and COU (0.5 mM).

Prepared sensing hydrogels were irradiated with X-ray tubes at dose rates of 0.7 Gy/min; radiation doses were 0.7 Gy, 1 Gy, 1.3 Gy, 1.7 Gy, 2 Gy, and 2.4 Gy, and three parallel samples were characterized at each dose. Subsequently, the fluorescence intensity of the radiated hydrogels were analyzed by a multimode plate reader. In the fluorescence intensity analysis, the excitation wavelength and emission wavelength were set as 340 nm and 460 nm, with a gain value of 100. The complete process is shown in Figure 1A.

#### 2.2.4. Devices and Measurements

All irradiation experiments were performed by energy irradiation of the samples using an X-ray tube with a voltage of 45 kV and a current of 0.4 mA (Dalian Tynman Technology Co., LTD, Dalian, China; TXR1010Ip50-50-A03). The dose rate of the ray tube was 0.7 Gy/min. In order to completely expose the sample to irradiation, a hydrogel sensor with a diameter of 8 mm was placed in the irradiation field of a 2.8 cm X-ray tube. The fluorescence intensity of irradiated samples was recorded using a multifunctional microplate reader (Tecan (Swiss) Trading Co., Ltd., Männedorf, Switzerland; INFINITE 200 PRO). Dynamic light scattering (DLS) of gold nanoparticles was obtained with a nanoparticle size and zeta potential analyzer (Malvern, Worcestershire, UK, Malvern Zetasizer Nano ZS9). TEM images of gold nanoparticles were measured by transmission electron microscopy (Thermo Fisher Scientific, WalthamUSA, FEI Talos F200X G2). UV-vis spectra of gold nanoparticles were measured using a UV-vis spectrophotometer (Shanghai Mapada Instrument Co., Ltd., Shanghai, China; ULH 1906005). The tensile experiments of agarose hydrogels were performed on an electronic universal testing machine (Shanghai Hesheng Instrument Technology Co., Ltd., Shanghai, China; HS-3000A).

## 3. Result and Discussion

### 3.1. Dose-Response and Principle of Hydrogel Sensor

Radiation induced fluoresce can be applied in radiotherapy to verify the absorbed dose of tissues that are exposed to radiation. Currently, it is known that water molecules produce ·OH when exposed to radiation, and COU can absorb the ·OH produced by the radiolysis of water to yield 7-HCOU with fluorescent effects [29] (Figure 1B). Consequently, a liquid sensor containing COU as the responsive material was created by dissolving COU in ultrapure water, and it was found to have an excellent dose-response in the low-dosage range (0–2.4 Gy) of fractionated radiation doses. (Figure 1C). As radiation therapy techniques continue to advance, however, liquid sensors as tools for point dose verification no longer match the accuracy and precision criteria of radiation therapy for three-dimensional absorbed dose and dose gradient measurements. At the same time, the composition of hydrogels is very similar to that of human tissue, which makes them particularly suitable as human tissue equivalents for the development of radiotherapy treatment plans. Therefore, one effective technique is to combine liquid sensors and hydrogels to form a hydrogel fluorometric dosimeter. However, this hydrogel sensor failed to realize linear response to low absorbed doses applied in the fractionated radiotherapy dose range (0–2.4 Gy) (Figure 1D).

The situation was improved after GNPs were introduced into the COU fluorometric hydrogel, for the reason that GNPs could increase the production of hydroxyl groups during irradiation. The mechanism and pathways that cause hydroxyl enhancement and fluorescence intensity are illustrated in Figure 1E [40]. In the first route, high-energy photons in the X-ray can directly interact with water, resulting in the radiolysis of water and the generation of ·OH; the GNPs can also participate in the process of radiolysis. When the X-rays interact with the GNPs in the primary phase, GNPs can absorb radiation energy to generate scattering electrons as well as Auger electrons at the same time, and the surface plasmon resonance (SPR) effect can further increase the radiation energy deposition on the GNPs. Then, the energy deposited is transmitted to the water, thus breaking the chemical bonds in the water molecules and producing ·OH. The last and most important route is known to occur in the structured water layer at the water–solid interface. As water is structured at the surface of GNPs, there is an additional hydrogen bond formed in the direction of dissociation, pulling along the H-OH bond and lengthening and weakening the intramolecular bond. As these bonds could already be strained, the energy injection could break them more easily. When X-rays interact with water, the energy produced by a series of excitations diffuses into the structured water layer, leading to the breakdown of the structured water and the production of more ·OH. Therefore, low-dose verification can be achieved by adding GNPs in this COU-based hydrogel. 

The top and front views of the sensing hydrogel are shown in Figure 1F. The sensing hydrogel measures the absorbed dose by the intensity of its fluorescence, which is affected by the excitation and emission wavelengths in the measurement. The emission wavelength of 7H-COU is known to be 460 nm, at which wavelength the fluorescence spectrum of the hydrogel sensor was measured. The fluorescence intensity was measured with an excitation wavelength of 340 nm (maximum excitation wavelength) and an emission wavelength of 460 nm (Figure 1G). The fluorescence intensity of the hydrogel sensor shows a linear relationship with its absorbed dose, ranging from 0 to 2.4 Gy with a linear correlation coefficient (r value) of 0.99308 (Figure 1H). The hydrogel sensor is therefore capable of dose verification in clinical fractionated radiotherapy.

### 3.2. Effect of GNP Concentration

**Figure 2:**
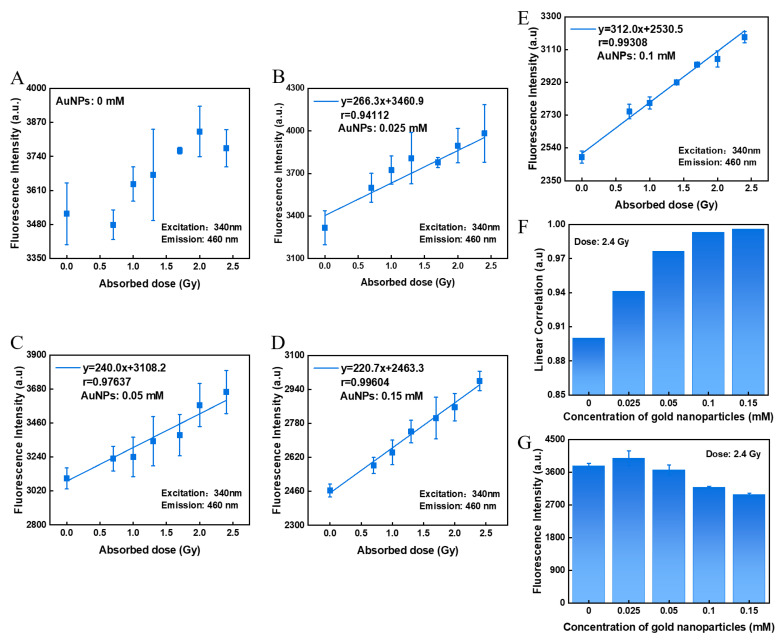
Effect of GNP concentration on the fluorescence intensity and dose-response of the hydrogel sensor. (**A**–**E**) The relationship between dose and fluorescence intensity of the hydrogel sensors with 0 mM, 0.025 mM, 0.05 mM, 0.15 mM, and 0.1 mM of GNPs. (**F**) The effect of GNP concentration on their linear fitting coefficients. (**G**) Effect of GNP concentration on fluorescence intensity at an irradiation dose of 2.4 Gy. In (**A**–**G**) the concentration of COU in the sensor is 0.5 mM and the mass fraction of agarose is 3 wt%.

We first evaluated the effect of GNPs on the dose-response of hydrogel sensors by adding different concentration of GNPs into the agarose, where 0 mM, 0.025 mM, 0.05 mM, 0.1 mM, and 0.15 mM of GNPs were added into a mixed hydrogel of COU and agarose, respectively. In the dose hydrogel without the addition of GNPs, there were very large error bars in the fluorescence-absorbed dose-response, and no obvious linear relationship between absorbed dose and fluorescence intensity was observed over a dose range of 0–2.4 Gy (Figure 2A). After the addition of GNPs to a mixed hydrogel of COU and agarose, the fluorescence intensity increased with increasing dose over the dose range of 0–2.4 Gy, with significantly smaller error bars. In Figure 2A–E, the linear relationship between dose and fluorescence intensity of the hydrogel sensor is shown for different concentrations of GNPs. It can be seen that the linear relationship between dose and fluorescence intensity of the hydrogel sensor gradually increases as the concentration of GNPs added increases (Figure 2F). When the concentration of GNPs in the hydrogel sensor reached 0.1 mM, the linear relationship between dose and fluorescence intensity had reached a dosimetric standard, after which the r value did not increase significantly by continuing to increase the GNP concentration. (Figure 2E,F). This is due to the fact that when the concentration of GNPs reaches 0.1 mM, the hydroxyl-enhancing effect at this dose is close to its maximum and the 7-HCOU produced by radiation reaches a plateau. As a result, it is recommended that the concentration of GNPs not be less than 0.1 mM in order to achieve optimal results.

However, as the concentration of GNPs increased, the fluorescence intensity of the hydrogel sensor detected first increased and then gradually decreased (Figure 2G). The enhanced fluorescence of the hydrogel sensor is due to the production of more hydroxyl groups in the presence of GNPs, and therefore, more 7-HCOU which can emit fluorescence. After that, the fluorescence intensity gradually decreased as the concentration of GNPs continued to increase due to the increased concentration of GNPs significantly decreasing the transparency of the hydrogel. The reduced transparency of the gel resulted in the scattering of light, which led to a decrease in the fluorescence intensity detected. At the same time, GNPs have a fluorescence-quenching effect on fluorescence [42], and as the concentration of GNPs increases, its fluorescence-quenching effect is enhanced, leading to a decrease in fluorescence intensity. When the concentration of GNP reaches 0.1 mM, there is already a good dose-response between 0–2.4 Gy. Therefore, a GNP concentration of 0.1 mM is an optimal concentration for low-dose verification.

### 3.3. Effect of Coumarin Concentration

**Figure 3:**
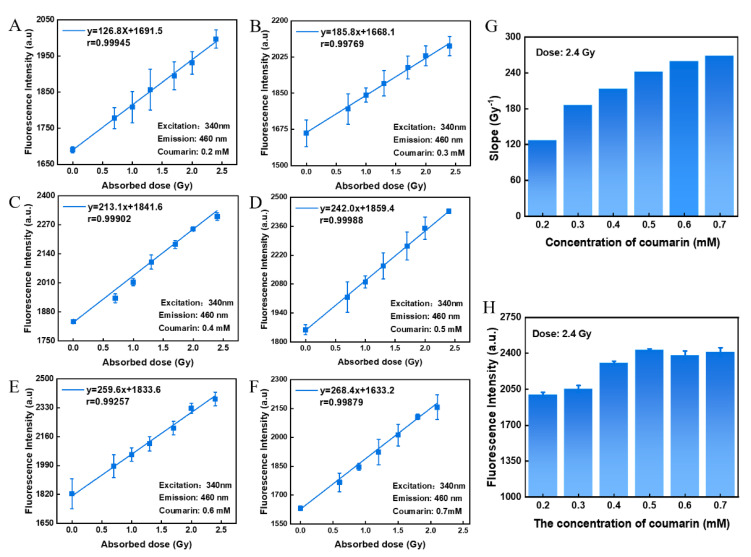
Effect of COU concentration on the fluorescence intensity and dose-response of hydrogel sensors. (**A**–**F**) The relationship between dose and fluorescence intensity with 0.2 mM, 0.3 mM, 0.4 mM, 0.5 mM, 0.6 mM, and 0.7 mM of COU. (**G**) The effect of COU concentration on the slopes of their linear fitting. (**H**) The effect of COU concentration on the fluorescence intensity of the hydrogel sensor. The concentration of GNPs was 0.1 mM and the mass fraction of agarose was 2 wt%.

The coumarin hydroxyl-capture method is known to be a common method for the quantitative determination of hydroxyl content [29,43,44]. It is mainly based on the interaction between COU and hydroxyl groups to form 7-HCOU with a fluorescent effect, which allows the fluorescence intensity of 7-HCOU to be measured to quantify the content of hydroxyl groups. Therefore, COU was used as a dose-responsive material for this hydrogel sensor, based on the fact that COU produces different levels of 7-HCOU under different radiation effects. Figure 3A–F show the linear relationship between absorbed dose and fluorescence intensity at different concentrations of COU. It was observed that as the concentration of COU in this hydrogel sensor increased, the slope of the linear relationship between dose and fluorescence intensity gradually increased, indicating that higher concentration of COU can capture a higher portion of ·OH before their consumption or recombination. In other words, the dose-response of this sensor is gradually enhanced with increasing COU concentration (Figure 3G). Furthermore, at first, the fluorescence intensity of this hydrogel sensor increased with an increasing COU concentration. However, this improvement failed to continue after the COU concentration increased to be larger than 0.5 mM (Figure 3H). It is shown that continuing to increase the concentration of COU beyond a concentration of 0.5 mM does not additionally increase the amount of 7-HCOU produced. Therefore, a COU concentration of 0.5 mM is optimal.

### 3.4. Effect of Agarose Mass Fraction

**Figure 4:**
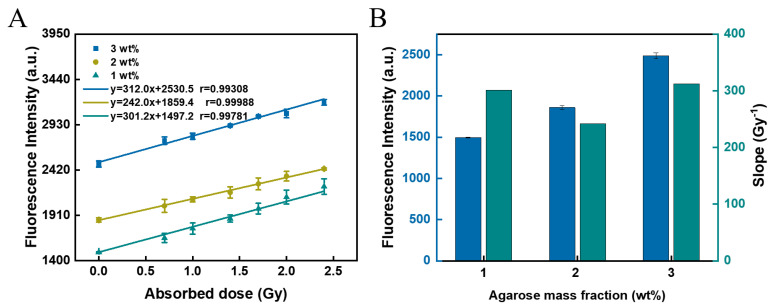
Effect of agarose mass fraction on the fluorescence intensity and linear relationship of hydrogel sensors. (**A**) Linear fitting of fluorescence intensity/absorbed dose curves with different agarose mass fractions (1 wt%, 2 wt%, and 3 wt%, respectively). (**B**) Effect of agarose mass fraction on fluorescence intensity and fitting slopes. The concentration of GNPs was 0.1 mM, and that of COU was 0.5 mM.

It is known that agarose melts into a liquid at high temperatures (>90 °C), adopts a random coil structure, and then cools to form a hydrogel at room temperature. The hydrogel forms a helical bundle by hydrogen bonding [45]. Hydrogel sensors with agarose mass fractions of 1 wt%, 2 wt%, and 3 wt% were prepared to explore the effect of agarose mass fraction on the dose-response of such hydrogel sensors. The fluorescence intensity of the hydrogel sensor that was not irradiated gradually increased with the mass fraction of agarose added (Figure 4A). This indicates the presence of a fluorescence background for this hydrogel sensor. However, this fluorescence background did not have an effect on the dose-response of the sensor. Meanwhile, when the mass fraction of agarose was 1 wt% and 2 wt%, the slope of the hydrogel sensor was relatively low, and the mechanical properties (Figure S2A, Supporting Information) could not even support the molding of hydrogel; when the mass fraction of agarose was 4 wt%, the brittleness of the agarose hydrogel increased, obviously, and it was easy to break (Figure S2B, Supporting Information); meanwhile, when the mass fraction of agarose was 3 wt%, there was a good dose-response between absorbed dose and fluorescence intensity (Figure 4B). Therefore, 3 wt% agarose was chosen as the basis for the hydrogel sensor.

### 3.5. Effect of GNP Size 

**Figure 5:**
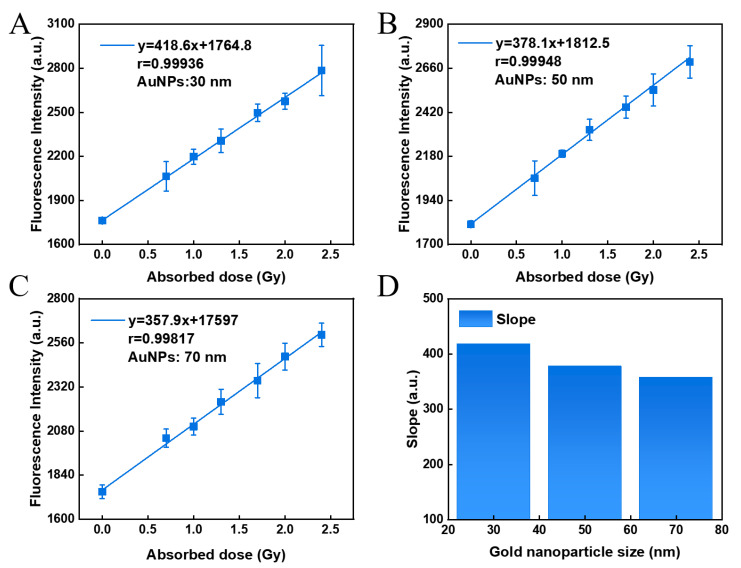
Dose-response of hydrogel sensor with different GNP sizes of 30 nm (**A**), 50 nm (**B**), and 70 nm (**C**), and their fitting slopes (**D**).

It is well known that the radiation enhancement of GNPs strongly depends on the size and concentration of nanoparticles as well as radiation energy [46]. Therefore, the effect of size of GNPs on dose-response should be considered. GNPs with sizes of 30 nm, 50 nm, and 70 nm were purchased and used as sensitizers in the mixed hydrogel of coumarin and agarose. As shown in Figure 5, the dose-response of GNPs with different sizes was observed. It was found that the slope of the dose–response curve of the hydrogel sensor was the largest when the GNP size was 30 nm, indicating that the dose-response of the sensor was slightly improved when the GNPs size get smaller.

### 3.6. Radiation Sensitivity of Hydrogel Sensors

The sensitivity of the hydrogel sensor was observed by the limit of detection (LOD). It is known that the limit of detection is calculated by the following equation [47]:

$${\mathrm{LOD}=3\;\sigma }_{B}/b$$

where *σ_B_* is the standard deviation of the fluorescence intensity to the unirradiated sample, and *b* is the slope of the dose–response curve. Calculations revealed that the LOD in the hydrogel sensor without the addition of gold nanoparticles was 1.5 Gy, which can meet the requirements of highly sensitive dose verifications. With the addition of gold nanoparticles, the LOD of hydrogel dosimeters was significantly decreased, which is listed in Table 1. In the best case, the LOD can reach 0.3 Gy, which is in the forefront, comparing to other previously reported hydrogel sensors [13].

## 4. Conclusions

In the current work, we prepared a hydrogel sensor with GNPs and COU as low-radiation dose-responsive materials using a very simple synthetic method, where the addition of GNPs plays a critical role in refining the linear correlation of its response. The hydrogel provides a method for dose-determination by measuring the fluorescence intensity of the generated 7-HCOU. The best dose-response was obtained in the hydrogel sensor with a concentration of 0.5 mM COU, 3 wt% agarose, and 0.1 mM GNPs, and obtained a detect-dose range of 0–2.4 Gy. All of these indicate the suitability of this approach in detecting radiation doses delivered during an individual fraction (typically 2 Gy) in fractionated radiotherapy. In conclusion, we believe that the GNP-enhanced radio-fluorogenic hydrogel dosimeter has great potential and provides a new route for clinical three-dimensional dose verification.

## Figures and Tables

**Table 1:** Limit of detection of the hydrogel sensor.

GNP Concentration (mM)	Beam	Linear Range (Gy)	LOD
0	X-ray	0–2.4	1.5
0.025	X-ray	0–2.4	1.3
0.05	X-ray	0–2.4	0.8
0.1	X-ray	0–2.4	0.3
0.15	X-ray	0–2.4	0.4

## Data Availability

Data are contained within the article.

## Supplementary Materials

The following supporting information can be downloaded at: https://www.mdpi.com/article/10.3390/polym14224841/s1, Figure S1: Relevant characterisation of the synthesised GNPs; Figure S2: Effect of mass fraction of agarose hydrogels on gel properties.
