# Supplementary material for: Gold-Nanoparticle-Enhanced Radio-Fluorogenic Hydrogel Sensor for Low Radiation Doses in Clinical Radiotherapy
Source: Polymers (Basel). 2022 Nov 10;14(22):4841. doi: 10.3390/polym14224841 (PMC9694710; doi:10.3390/polym14224841)
Supplement: Supplementary file 1 [file polymers-14-04841-s001.zip › polymers-2019310-supplementary.pdf]

Supporting Information

Gold Nanoparticle-Enhanced Radio-Fluorogenic Hydrogel Sensor for Low Radiation Dose in Clinical Radiotherapy

Xingyu Dong[†], Yuan Tian[†], Fengqing Wang, Chong Chen, Yunlong Wang*, and Jun Ma*

College of Materials Science and Technology, Nanjing University of Aeronautics and Astronautics Nanjing 211106, P. R. China.

Correspondence: authors: wylong@nuaa.edu.cn; junma@nuaa.edu.cn

[†]These authors contributed equally to this work.

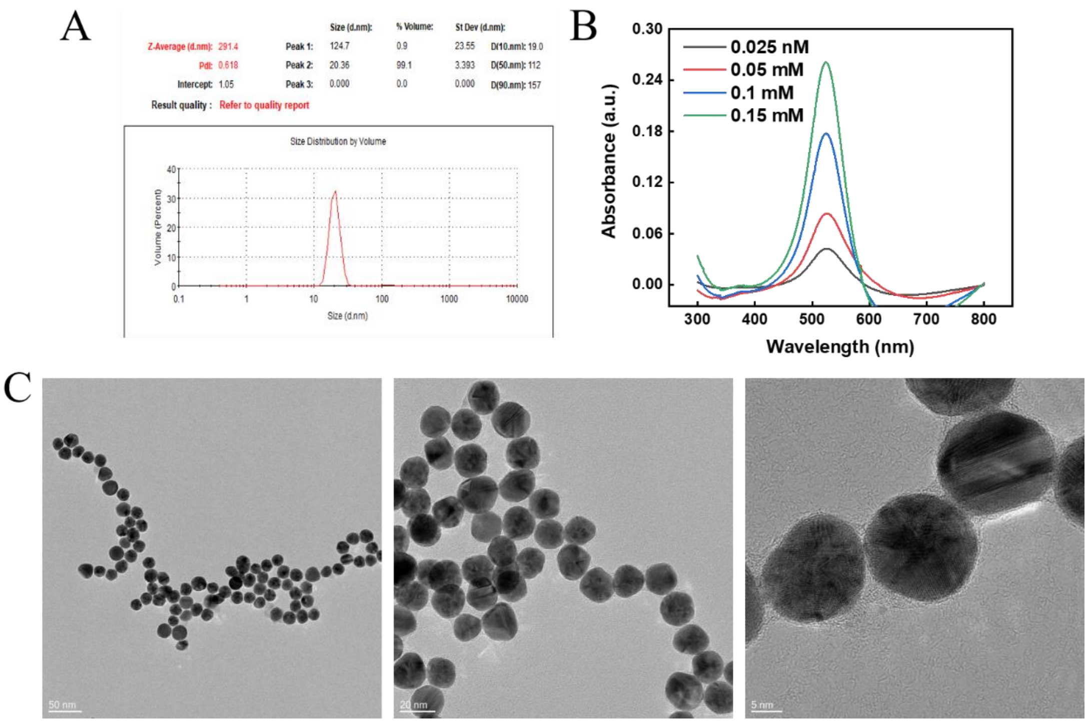

Supplementary Figure S1. Relevant characterisation of the synthesised GNPs. A: Dynamic light scattering from GNPs. B: UV-vis on spectra of different concentrations of GNPs. C: TEM image of GNPs.

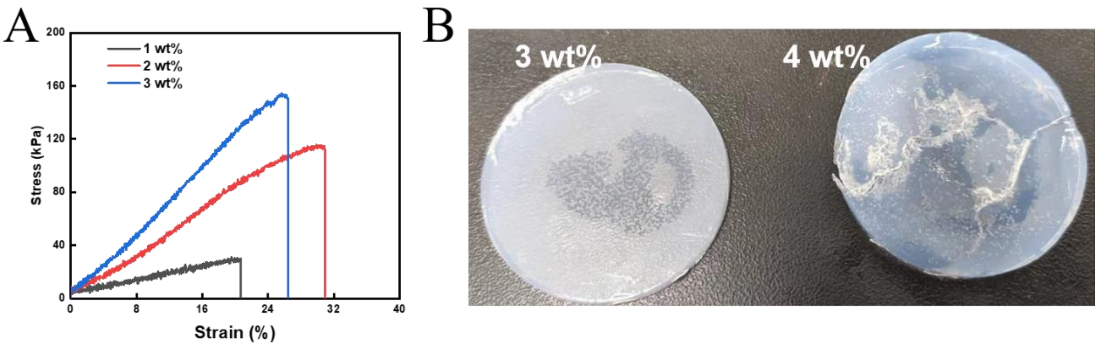

Figure S2. Effect of mass fraction of agarose hydrogels on gel properties. A: Stress-strain curve of hydrogels with different mass fractions. B: The digital photos of gels with a mass fraction of 3% and 4% after demoulding, respectively.
